# Emergency tips recanalisation and gastroesophageal varices embolisation with an ethylene vinyl alcohol copolymer agent (Squid) and detachable coils

**DOI:** 10.1186/s41747-020-00196-9

**Published:** 2020-12-10

**Authors:** Massimo Venturini, Luigi Augello, Carolina Lanza, Marco Curti, Andrea Coppola, Filippo Piacentino, Francesco De Cobelli

**Affiliations:** 1 Department of Diagnostic and Interventional Radiology, Circolo Hospital, Viale Borri 57, 21100 Varese, Italy; 2 Insubria University, Varese, Italy; 3 Department of Clinical and Experimental Radiology, San Raffaele Scientific Institute, Milan, Italy; 4 Department of Radiology, European Institute of Oncology, Milan, Italy; 5 Vita-Salute San Raffaele University, Milan, Italy

**Keywords:** Embolization (therapeutic), Esophageal and gastric varices, Ethylene-vinyl alcohol copolymer, Hypertension (portal), Portosystemic shunt (transjugular intrahepatic)

## Abstract

Transjugular intrahepatic portosystemic shunt (TIPS) is currently indicated as first therapeutic option in the main complications of portal hypertension, including bleeding gastroesophageal varices and refractory ascites. In case of bleeding gastroesophageal varices, an adjuvant embolisation within TIPS can be useful to prevent rebleeding. In the present technical note, the management in emergency of a patient with haemorrhagic shock due to bleeding gastroesophageal varices and occluded TIPS is reported. TIPS recanalisation with an adjunctive stent and high-pressure balloon angioplasty and gastroesophageal varices embolisation using detachable coils and a non-adhesive liquid embolic agent were performed during the same emergent procedure. After the procedure, clinical stabilisation of the patient was achieved, with blood transfusions suspension and Blakemore tube removal. At 6 months, regular TIPS patency at colour Doppler and no rebleeding episodes were recorded. To our knowledge, whilst coils are routinely used for varices embolisation, non-adhesive liquid embolic agents have been never mentioned. Liquid embolic agents seem to provide a stable plug strengthening the embolising action of the coils. Further studies involving a cohort of patients with long-term follow-up will be necessary to confirm whether this association can be more effective than coils alone in gastroesophageal varices embolisation.

## Key points

- Bleeding gastroesophageal varices are a typical complication of portal hypertension usually requiring transjugular intrahepatic portosystemic shunt (TIPS) in election or in emergency.
- Non-adhesive liquid embolic agents as ethylene-vinyl alcohol copolymers are routinely used in cerebral field, less often in abdominal district.
- Coil embolisation of bleeding gastroesophageal varices during the TIPS placement or in case of occluded TIPS is a possible adjunctive procedure.
- The use of a non-adhesive liquid embolic agent associated with coils can potentially strengthen the embolic action probably allowing a more permanent occlusion of gastroesophageal varices than coils alone.

## Background

Portal hypertension remains the main complications of cirrhosis. Transjugular intrahepatic portosystemic shunt (TIPS) represents the main therapeutic option in case of refractory ascites and bleeding from gastroesophageal varices, in particular when medical or endoscopic treatments fail [1, 2].

An adjuvant embolisation of gastroesophageal varices within TIPS procedure can be useful to prevent a rebleeding. Coil embolisation is routinely used, although alternative embolic agents or devices, such as glue or plugs [3–5], were also used in gastroesophageal varices embolisation. Advantages of detachable coils, nowadays less expensive than in the past, are their marked radiopacity and their capability to be removed and replaced when necessary, whilst their major limit is the achievement of a complete and permanent occlusion [6]. Non-adhesive, ethylene-vinyl alcohol (EVOH) liquid embolic agents mixed with micronised tantalum powder and dissolved in dimethyl sulfoxide (DMSO), as Onyx and Squid, were mainly used in the cerebral district [7, 8]. However, they have recently also used in abdominal diseases, alone as in endoleak embolisation [9, 10] or associated with coils for treating splenic [11] or renal aneurysms [12]. Squid, more recently introduced than Onyx, associated with coils seems to provide a more stable and totally occlusive cast to prevent recanalisation. In the present technical note, an emergency TIPS recanalisation with bleeding gastroesophageal varices embolisation using Squid and detachable coils is presented.

## Case report

**Fig. 1:**
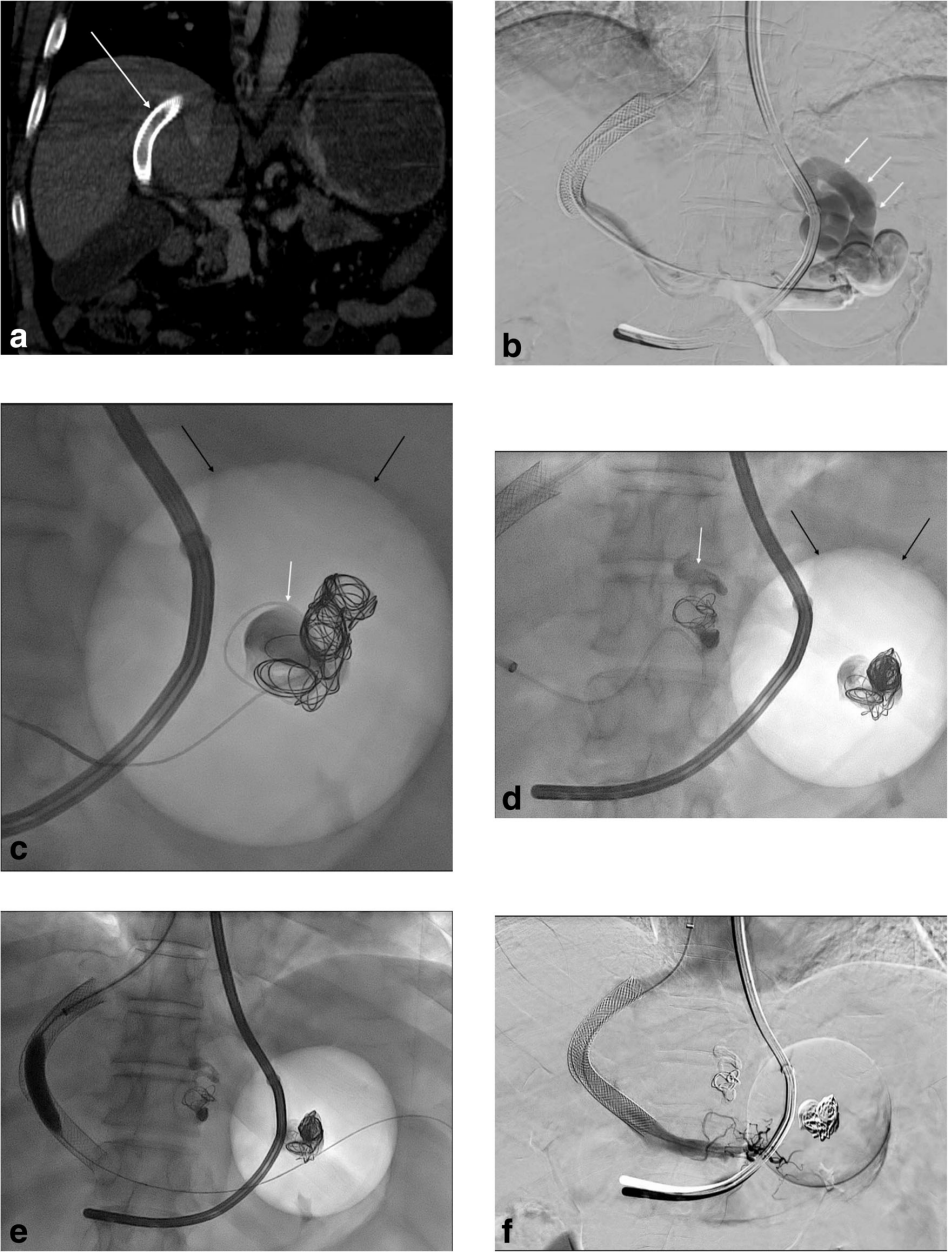
**a** Contrast-enhanced computed tomography in emergency in a cirrhotic patient with haematemesis shows an occluded transjugular intrahepatic portosystemic shunt (TIPS) (white arrow). **b** Portography after TIPS recanalisation shows large gastric varices (white arrows). **c** Embolisation with an ethylene-vinyl alcohol (EVOH) liquid embolic agent (white arrow) and detachable coils of the gastric varices through a coaxial microcatheter. A Blakemore tube with inflated balloon in the stomach is evident (black arrows). **d** After gastric varices embolisation in the projective correspondence of the Blakemore tube (black arrows), also oesophageal varices were embolised with liquid embolic agent (white arrow) and detachable coils. **e** After gastroesophageal varices embolisation and an overlapped second stent placement, TIPS recanalisation was completed with high-pressure balloon angioplasty. **f** Final portography shows a quick opacification of the TIPS and the right atrium as good function of the portosystemic shunt without more evidence of the gastroesophageal varices

The management of a 58-year-old man with post-hepatitis C advanced cirrhosis, mild ascites and massive haematemesis come to the emergency department of our hospital is referred. The patient presented in our emergency department with massive haematemesis, hypotension (blood pressure 90/60 mmHg), tachycardia (110 beats per minute) and anaemia (haemoglobin level 9 mg/dL). Haemodynamic instability of the patient was treated by blood transfusions and by Blakemore tube placement in the stomach. An emergency contrast-enhanced computed tomography (CT) showed large gastroesophageal varices and an occluded TIPS (Fig. 1a) placed 3 years ago at another hospital. A TIPS recanalisation with gastroesophageal varices embolisation was planned in emergency setting. The hemodynamically stabilised patient signed a specific institutional procedure-related consent form that covers retrospective observational studies. The procedure was performed by an experienced (> 20 years) interventional radiologist (M.V.) during patient conscious sedation.

After the placement of a 7-F introducer in the right jugular vein, the occluded TIPS was crossed using a 0.035-in. hydrophilic guidewire (Terumo, Tokyo, Japan) and a 4-F Headhunter catheter (Cordis, Miami Lakes, FL, USA). Portal pressure was 36 mmHg. The preliminary portography showed large gastroesophageal varices (Fig. 1b). First gastric varices (Fig. 1c), then oesophageal varices (Fig. 1d) were catheterised with a coaxial microcatheter (1.9 Carnelian, Tokai, Sarayashiki Taraga Kasugay City, Japan) and progressively embolised with detachable coils (18–20-mm diameter, 30–50-cm length; Interlock, Boston Scientific Corporation, Marlborough, MA, USA) and an (EVOH) copolymer agent (Squid-Peri 34, Emboflu, Gland, Switzerland). EVOH liquid embolic agent was shacked for 15–20 min and, before administration, the microcatheter dead space was filled with DMSO. Squid was then slowly injected to minimise DMSO-related pain due to its endothelial toxicity, progressively retracting the microcatheter to avoid its entrapment. No pain was recorded during Squid administration. After gastroesophageal varices embolisation, a self-expandable bare metal stent (12 mm diameter, 8 cm length; Wallstent, Boston Scientific Corporation, Marlborough, MA, USA) was overlapped to the previous stent and finally a balloon-angioplasty (10 mm diameter, 4 cm length, 18 atm insufflation pressure) of the whole TIPS was performed (Fig. 1e). Portal pressure at the end of the procedure was 24 mmHg. The final portography showed a quick opacification of the TIPS and the right atrium without gastroesophageal varices evidence, as good function of the portosystemic shunt (Fig. 1f). Contrast material administered during the procedure was about 60 mL of Iopromide (Ultravist 370, Bayer HealthCare, Berlin, Germany). After the procedure, clinical stabilisation of the patient was achieved, blood transfusions were suspended and Blakemore tube was removed. At discharge 6 days after the procedure, haemoglobin level was 11.4 mg/dL. No rebleeding episodes were recorded during 6 months of follow-up. Colour Doppler ultrasound at 24 h and at 1 and 6 months confirmed the TIPS patency.

## Discussion

Venous embolisation with an EVOH liquid embolic agent associated with *n*-butyl-cyanoacrylate was previously described for occlusion of portal branches in preoperative portal vein embolisation [13]. Squid use as embolic agent in various abdominal diseases [10] and its association with detachable coils in visceral artery aneurysms were recently reported [11, 12]. In the present case, detachable coils were first placed to provide the scaffold, subsequently compacted by Squid 34, the most viscous formulation, achieving a stable and occlusive plug. EVOH liquid embolic agent can strengthen the embolising power of the coils. The two best known non-adhesive EVOH liquid embolic agents are Onyx and Squid, both available in different formulations characterised by variable viscosity. They are similar but Squid, marketed more recently, is also available in the less viscous formulation which spreads more distally and may be useful in selected cases, as for example to treat arteriovenous malformations [8, 14]. Another advantage of Squid over Onyx is the reduced percentage (less 30%) and smaller size of tantalum particles conditioning fewer metallic artefacts on CT images [15, 16]. Compared to adhesive liquid embolic agents as glue, Squid and Onyx are more expensive but can provide a more predictable formulation-dependent distribution, resulting in more homogeneous casts. Moreover, the risk of accidental complications due to non-target vessel embolisation is probably higher using glue than EVOH embolic agents: glue spreads in a flow-dependent manner and can potentially be displaced during microcatheter retraction, if stuck to its tip [17]. This risk can increase in case of a retraction of a microcatheter for a long distance without the protection of a standard angiographic catheter as for example in the case of a type 2 endoleak embolisation performed through the Riolano arch [18]. A disadvantage of non-adhesive liquid embolic agents is the need of DMSO, potentially endotheliotoxic and unnecessary using glue.

Before Squid infusion, DMSO (not radiopaque) was slowly injected to minimise the possible pain due to its endothelial toxicity. Usually, in case of DMSO infusion in a large vascular bed, such as gastroesophageal varices, procedures are well tolerated without need for pain medication [19], as in our case. After gastroesophageal varices embolisation, TIPS was prolonged in portal vein with a second bare metal stent overlapped with the first previously placed stent and finally a high-pressure balloon angioplasty was performed.

Summarising, in our preliminary experience, simultaneous TIPS recanalisation and gastroesophageal embolisation with an EVOH liquid embolic agent and coils was successfully performed in emergency, with good clinical mid-term outcome. Further studies involving cohorts of patients with long-term follow-up are needed to validate the association of EVOH liquid embolic agents and coils in gastroesophageal varices embolisation.

## Data Availability

Data and materials can be provided on request.

## Abbreviations

CT: Computed tomography
DMSO: Dimethyl sulfoxide
EVOH: Ethylene-vinyl alcohol
TIPS: Transjugular intrahepatic portosystemic shunt
